# The Interstitial Interface within the Renal Stem/Progenitor Cell Niche Exhibits an Unique Microheterogeneous Composition

**DOI:** 10.3390/ijms140713657

**Published:** 2013-06-28

**Authors:** Will W. Minuth, Lucia Denk

**Affiliations:** Department of Molecular and Cellular Anatomy, University of Regensburg, Regensburg D-93053, Germany; E-Mail: lucia.denk@vkl.uni-regensburg.de

**Keywords:** kidney, transmission electron microscopy, stem/progenitor cell niche, interstitial interface, extracellular matrix, cupromeronic blue, ruthenium red, tannic acid

## Abstract

Repair of parenchyma by stem/progenitor cells is seen as a possible alternative to cure acute and chronic renal failure in future. To learn about this therapeutic purpose, the formation of nephrons during organ growth is under focus of present research. This process is triggered by numerous morphogenetic interactions between epithelial and mesenchymal cells within the renal stem/progenitor cell niche. Recent data demonstrate that an astonishingly wide interstitial interface separates both types of stem/progenitor cells probably controlling coordinated cell-to-cell communication. Since conventional fixation by glutaraldehyde (GA) does not declare in transmission electron microscopy the spatial separation, improved contrasting procedures were applied. As a consequence, the embryonic cortex of neonatal rabbit kidneys was fixed in solutions containing glutaraldehyde in combination with cupromeronic blue, ruthenium red or tannic acid. To obtain a comparable view to the renal stem/progenitor cell niche, the specimens had to be orientated along the cortico-medullary axis of lining collecting ducts. Analysis of tissue samples fixed with GA, in combination with cupromeronic blue, demonstrates demasked extracellular matrix. Numerous braces of proteoglycans cover, as well, the basal lamina of epithelial stem/progenitor cells as projections of mesenchymal stem/progenitor cells crossing the interstitial interface. Fixation with GA containing ruthenium red or tannic acid illustrates strands of extracellular matrix that originate from the basal lamina of epithelial stem/progenitor cells and line through the interstitial interface. Thus, for the first time, improved contrasting techniques make it possible to analyze in detail a microheterogeneous composition of the interstitial interface within the renal stem/progenitor cell niche.

## 1. Introduction

Acute and chronic renal failure in humans are worldwide on an increase, possibly reaching epidemic proportions in the future [1]. Major causes for the constant increase are, to date, unclear. The conventional kinds of therapy are either hemodialysis over years or, if available, transplantation of a donor organ [2,3]. However, due to known limitations of these therapies an increasing number of research projects are dealing, in the last few years, with the question if implantation of stem/progenitor cells will be an effective therapeutic alternative in the future. Despite first efforts and experiences, a breakthrough, including a therapeutic success, is until now not in sight. Up to date unsolved problems are the need of suitable stem/progenitor cells, a limited survival of implanted cells and an ineffective technique for a site-specific seeding in diseased parenchyma [4,5]. Due to that background it is obvious that intense basic research has to be performed about capacity of stem/progenitor cells to repair diseased parenchyma in parallel to its physiological development in the embryonic kidney [6].

During organ development, survival of stem/progenitor cells and initial formation of parenchyma is guaranteed within the unique structural environment of the renal stem/progenitor cell niche [7]. As a consequence, learning from this surrounding may help to find in analogy innovative biomedical tools to facilitate the survival of implanted stem/progenitor cells, and to support initial steps of regeneration. However, to date, the knowledge about development of renal parenchyma is rather rudimentary. While numerous publications exist dealing with the formation of the kidney anlage and the induction of individual nephrons, sound information about successive development such as tube formation, polarization, spatial growth and physiological maturation of individual segments is limited [8]. The situation is further complicated as renal parenchyma does not derive from a single stem/progenitor cell lineage, but originates by interactions between nephrogenic mesenchymal and epithelial stem/progenitor cells. Both types of cells are facing each other within the renal stem/progenitor cell niche [9].

Nephron development starts by a strict temporospatial program that controls a collecting duct (CD) ampulla tip (originally derived from the ureteric bud) to divide dichotomously [10]. The resulting bifurcation is shunting contained epithelial stem/progenitor cells in the correct position to neighboring mesenchymal stem/progenitor cells so that an exchange of morphogenetic molecules such as GDNF, Wnt11, c-Ret, BMP and FGFRI/II can take place [11]. The molecular actions lead first to a separation of few mesenchymal stem/progenitor cells out of the cap condensate and then to an aggregation. The ensuing mesenchymal-to-epithelial transition produces successively a Comma-, Pine Cone- and a S-shaped body as morphological signs of proceeding nephron formation [12]. While primary steps of nephron development are under way, fully separated within the interstitium, the cortical wing of the arising S-shaped body is fusing with the lateral aspect of the CD ampulla. During this process the developing nephron is functionally connected with the CD tubule.

The outer cortex of the developing kidney, including the renal stem/progenitor cell niches, is not provided by an intact microvascular system [13]. It is presumed that the exchange of morphogenetic molecules takes place via paracrine secretion and consequent diffusion. For avoiding loss of bioactive information one consequently expects that the concentration of secreted morphogenetic molecules is high and that the distance for diffusion is short to reach the target [14]. This again implies that a close contact should exists between both kinds of stem/progenitor cells. However, the contrary is true. Analysis of human or rabbit kidney demonstrates that epithelial stem/progenitor cells within a CD ampulla are separated from mesenchymal stem/progenitor cells by a bright interstitial interface [15].

Transmission electron microscopy illustrates, after traditional fixation of specimens in glutaraldehyde, that a wide but not very noticeable interstitial interface exists between epithelial and mesenchymal stem/progenitor cells [15]. Further filigree, but intact projections from mesenchymal cells, are crossing the interstitial interface, indicating that it is not an artificial gap but a highly structured connection [16]. In addition, a remarkable microarchitecture can be recognized, when contained extracellular matrix is demasked by fixation of specimens in glutaraldehyde containing cupromeronic blue, ruthenium red or tannic acid. All of these new findings demonstrate that cells within the renal stem/progenitor cell niche are not accidentally distributed, but are kept in a sustainable order. Thus, to gather more detailed information about structural compounds of the interstitial interface within the renal stem/progenitor cell niche, the present investigation was performed.

## 2. Results and Discussion

### 2.1. Vertical Sight of the Renal Stem/Progenitor Cell Niche

**Figure 1:**
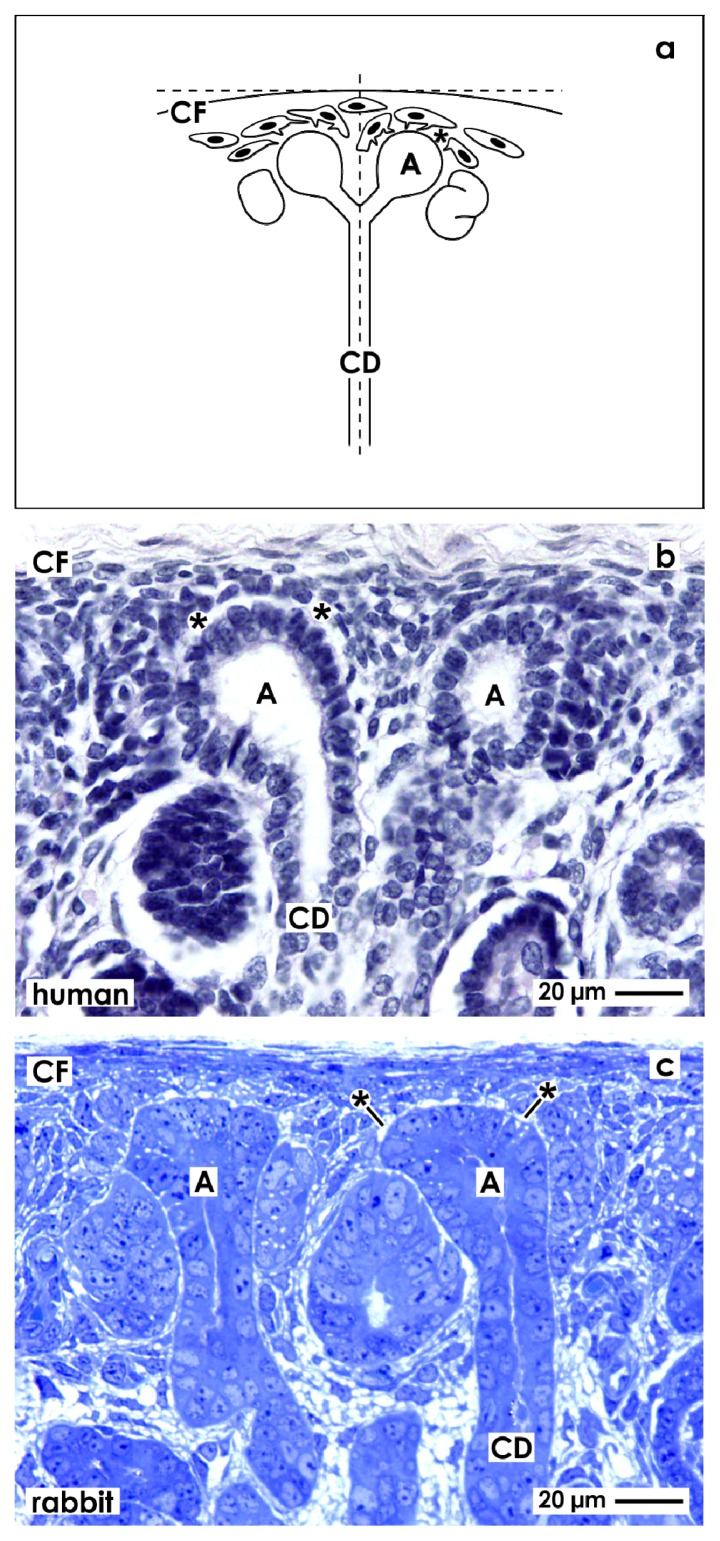
Comparable view to the stem/progenitor cell niche needs accurate orientation of the renal cortex for histological sectioning. (**a**) Schema depicts that a vertical view is obtained by cutting in parallel to the lumen of lining collecting ducts (CD) and perpendicular to the Capsula fibrosa (CF). In addition, in human fetal (**b**) as in rabbit neonatal (**c**) kidney can be recognized under the light microscope that epithelial stem/progenitor cells are contained in the CD ampulla (A) tip. Mesenchymal stem/progenitor cells are found beyond the organ capsule and surround the basal aspect of the CD ampulla. Both types of stem/progenitor cells are separated by the interstitial interface (asterisk).

For orientation, the renal stem progenitor cell niche is found in the outer cortex and in close vicinity to the Capsula fibrosa (CF). It exhibits remarkable morphological and site-specific characteristics (Figure 1a). Epithelial stem/progenitor cells are enclosed within the CD ampulla tip, while neighboring mesenchymal stem/progenitor cells are separated by the interstitial interface. Experiences from the last years demonstrate that the morphological peculiarities of this interface only can be investigated, when a clear orientation of the tissue bloc is performed before sectioning by the microtome takes place.

To obtain a comparable view the parenchyma is orientated along the cortico-medullary axis and in parallel to the lumen of lining collecting ducts (CD). As a consequence, all of the following micrographs show this perspective so that comparisons between the renal stem/progenitor cell niche in different experimental series can be performed. For clear identification, the plasma membrane at the tip of a CD ampulla is indicated by a cross (+), when illustrations are shown made by transmission electron microscopy.

### 2.2. Light Microscopical View to the Renal Stem/Progenitor Cell Niche

Formation of intact renal parenchyma is depending on the one hand on reciprocal morphogenetic interactions [9–11] and on the other hand on the exact spatial orientation of cells within the renal stem/progenitor cell niche [6]. This can be seen on sections through the outer cortex and always in close vicinity to the organ capsule of fetal human (Figure 1b) or neonatal rabbit (Figure 1c) kidney. The ureteric bud derived tip of a CD ampulla encloses epithelial stem/progenitor cells. As recognized on vertical sections, in the space between the inner layer of the organ capsule and the tip of a CD ampulla (14 to 16 μm), two to three layers of mesenchymal stem/progenitor cells are found earlier described as cap condensate. The surface view in light microscopy further illustrates that both types of stem/progenitor cells do not contact each other but are separated by an astonishingly wide interstitial interface lining in parallel to the basal lamina of a CD ampulla tip.

### 2.3. Electron Microscopical View to the Renal Stem/Progenitor Cell Niche

#### 2.3.1. Traditional Fixation with Glutaraldehyde (GA)

**Figure 2:**
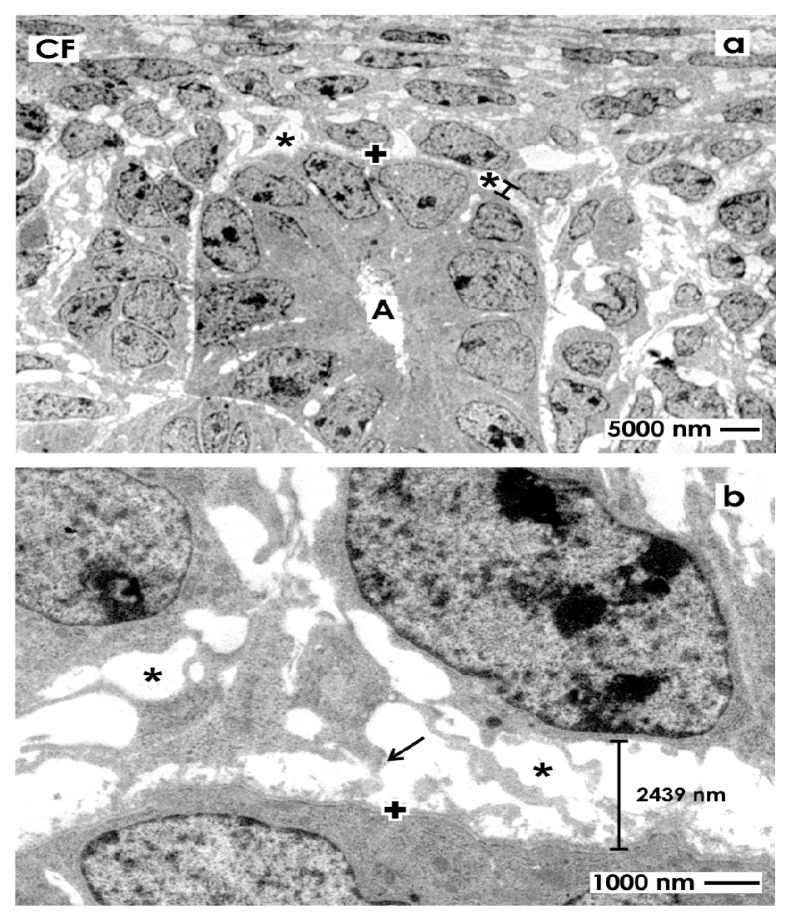
Transmission electron microscopy of the renal stem/progenitor cell niche in neonatal rabbit kidney after fixation in glutaraldehyde (GA). (**a**) Low magnification depicts that epithelial stem/progenitor cells are enclosed by the basal lamina (+) at the collecting ducts (CD) ampulla (A) tip. The soma of mesenchymal stem/progenitor cells is separated from epithelial cells by the interstitial interface (asterisk/spacer); (**b**) Higher magnification shows that epithelial and mesenchymal stem/progenitor cells are kept in this typical case at a distance of 2439 nm by the bright interstitial interface.

To better illustrate the omnipresent interstitial interface within the renal stem/progenitor cell niche, specimens of embryonic parenchyma were traditionally fixed with glutaraldehyde (GA) to investigate ultrathin sections under low magnification in transmission electron microscopy (Figure 2).

A surface view shows typical features within the renal stem/progenitor cell niche. Integrated within the tip of a CD ampulla (A), epithelial stem/progenitor cells are found (Figure 2a). The basal aspect of the ampulla is surrounded by mesenchymal stem/progenitor cells. Both types of cells are separated by the bright interstitial interface. Higher magnification illustrates that mesenchymal stem/progenitor cells send out filigree cell projections, which cross the interstitial interface to contact at irregular distances the basal lamina at the CD ampulla tip (Figure 2b). Further, the distance between the basal lamina at the CD ampulla tip, mesenchymal stem/progenitor cells and their crossing projections is about 2400 nm, appears bright and exhibits no recognizable extracellular matrix. Finally, in all of the cases was seen that the interstitial interface within the renal stem/progenitor cell niche keeps epithelial in distinct distance to mesenchymal cells. As a consequence, clustering or close contacts between cells was never observed.

**Figure 3:**
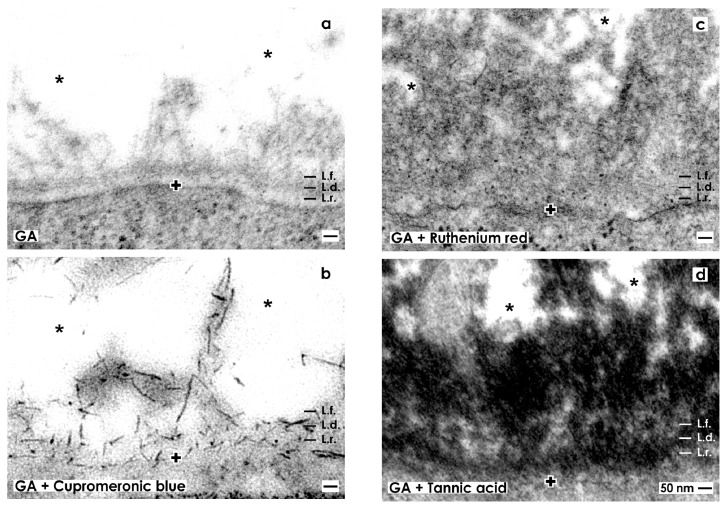
Transmission electron microscopy of the renal stem/progenitor cell niche. The basal lamina enclosing epithelial stem/progenitor cells at the CD ampulla tip is labeled by a cross (+). An astonishingly wide interstitial interstitial interface (asterisk) keeps mesenchymal stem/progenitor cells in distance. (**a**) Specimens fixed by GA show a basal lamina consisting of a lamina rara (L.r.), a lamina densa (L.d.) and an extended lamina fibroreticularis (L.f.). The interstitial interface is bright. (**b**) Samples fixed by GA including cupromeronic blue exhibit that buckles of proteoglycans are incorporated in the basal lamina. Further lining projections from mesenchymal stem/progenitor cells are covered by proteoglycans. (**c**) Specimens fixed by GA including ruthenium red illustrate that a broad band of heterogeneously composed label is established along the basal lamina and within the interstitial interface. (**d**) Specimens fixed by GA including tannic acid demonstrate that a broad band of heterogeneously composed label is present along the basal lamina and within the interstitial interface.

Under high magnification specimens fixed in traditional GA depict a consistently developed basal lamina covering epithelial stem/progenitor cells within the tip of a CD ampulla (Figure 3a). The basal lamina illustrates a clearly visible lamina rara (L.r.), lamina densa (L.d.) and tiny fibers of the lamina fibroreticularis (L.f.) as was earlier described [15]. Projections of mesenchymal stem/progenitor cells searching contact with the lamina fibroreticularis can be barely recognized using traditional GA for fixation. In addition, collagen fibers exhibiting a certain periodicity were not detected.

**Figure 4:**
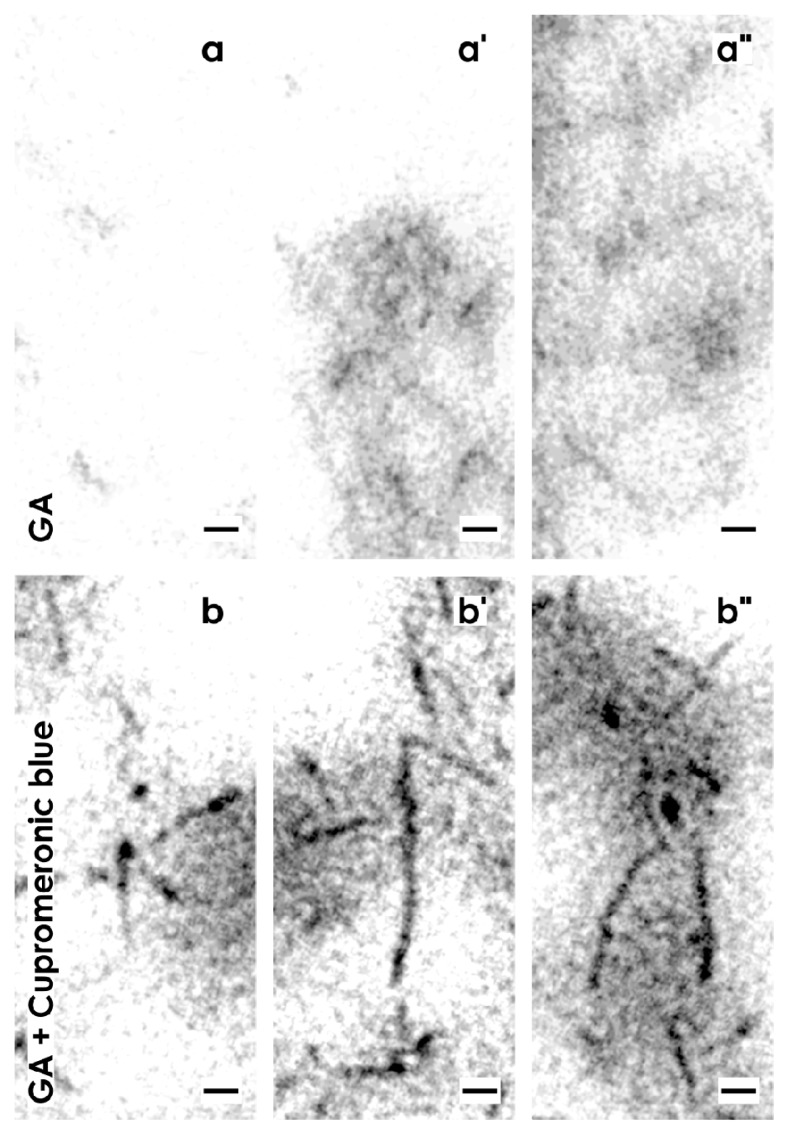

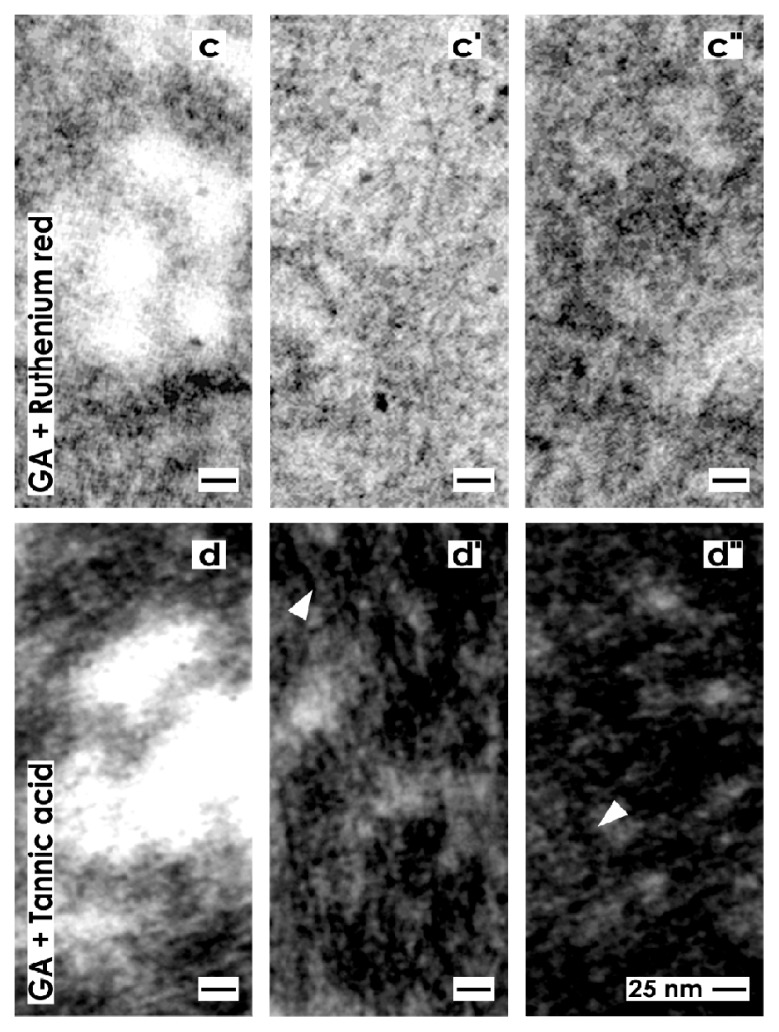
Transmission electron microscopy of the renal stem/progenitor cell niche. (**a**) Specimens fixed by GA depict in the interstitial interface in bright areas tiny fibers of extracellular matrix. (**a**′) In neighboring regions fibers are bundled. (**a**″) In other areas the same fibers form a woven network. (**b**) Specimens fixed by GA including cupromeronic blue show that buckles of proteoglycans occur as a lengthwise body and a more or less round head. (**b**′) They show a length of more than 100 nm. (**b**″) A buckle of proteoglycan can be linked to the next so that longitudinal chains arise. In addition, buckles are observed that are linked on different sites so that a three dimensional network arises. (**c**) Specimens fixed by GA including ruthenium red illustrate translucent bundles of extracellular matrix traversing the interstitial interface. In their neighborhood, areas are present with light grey (**c**′) and dark grey (**c**″) but fine-grained extracellular matrix. (**d**) Specimens fixed with GA including tannic acid demonstrate that bright but also intensively labeled areas exist at the interstitial interface. (**d**′,**d**″) The label for tannic acid is not accidently distributed, but exhibits a remarkable microarchitecture on fibrillar structures in form of solid particles.

High magnification in TEM shows a microheterogeneous composition of the interstitial interface. In bright areas tiny fibers of extracellular matrix can be detected (Figure 4a). In neighboring regions fibers were bundled (Figure 4a′). In other areas the same fibers formed a woven network (Figure 4a″).

The wide interstitial interface within the renal stem/progenitor cell niche observed after traditional fixation of specimens in GA (Figures 2,3a,4a–a″) might be caused by fluid pressure or masked extracellular matrix. As such suspected structures are not visible after traditional fixation, improved contrasting with cupromeronic blue, ruthenium red and tannic acid was performed.

#### 2.3.2. Contrasting with GA Including Cupromeronic Blue

Fixation of specimens with GA containing cupromeronic blue illustrates that buckles of proteoglycans are incorporated in the basal lamina at the CD ampulla tip (Figure 3b). Especially, the basal plasma membrane and lamina fibroreticularis are bordered by proteoglycans. Further, each of the lining projections from mesenchymal stem/progenitor cells is covered by buckles of proteoglycans. Thus, applying cupromeronic blue, contrasting (Figure 3b) the contact zone between cell projections and the lamina fibroreticularis, shows other features than was seen after traditonal fixation in GA (Figure 3a). Surprisingly, the interstitial interface between the lamina fibroreticularis and mesenchymal cell projections appears bright and does not reflect any fibers of extracellular matrix (Figure 3b).

Very high magnifications in TEM illustrate that buckles of proteoglycans consist, in the typical case, of a lengthwise body and a more-or-less round head (Figure 4b). The buckles illustrate that proteoglycans can exhibit a length of more than 100 nm (Figure 4b′). At their endings they can be linked to the next one so that longitudinal chains arise. Finally, buckles of proteoglycans are observed, which are linked on different sites so that a three dimensional network arises (Figure 4b″).

#### 2.3.3. Contrasting with GA Including Ruthenium Red

Fixation of specimens with GA containing ruthenium red shows that the basal lamina bordering the interstitial interface appears completely different as compared to previous series (Figure 3a,b). Although, the typical three-laminar structure of the basal lamina at the CD ampulla tip consisting of a lamina rara, lamina densa and lamina fibroreticularis cannot be recognized anymore after ruthenium red label (Figure 3c). Instead, a broad band of cloudy ruthenium red label is established along the basal lamina. However, it reveals a heterogeneous composition.

Very high magnifications in TEM illustrate, at the lamina fibroreticularis, translucent bundles of extracellular matrix traversing the interstitial interface (Figure 4c). As these translucent fibers do not exhibit a repeating period, they cannot be ascribed to a certain type of collagen. In their neighborhood, alternating areas exists containing light grey (Figure 4c′) and dark grey (Figure 4c″) but in all cases fine-grained extracellular matrix. These structures are not identical to extracellular matrix found after fixation with GA (Figure 4a–a″) or GA in combination with cupromeronic blue (Figure 4b–b″). The results speak for a heterogeneous structural composition of the interstitial interface within the renal stem/progenitor cell niche, which can be visualized by ruthenium red label.

#### 2.3.4. Contrasting with GA Including Tannic Acid

Fixation with GA containing tannic acid exhibits an intense label at the basal lamina of the CD ampulla tip (Figure 3d). Surprisingly, the complete basal lamina is covered by a broad but more-or-less electron-dense coat and similar to as it was detected after fixation with GA containing ruthenium red (Figure 3c). However, the label for tannic acid is much more intensive than the label with ruthenium red. In addition, areas at the interstitial interface exist, which are bright and consequently not labeled by tannic acid. This result speaks in favor of a stain-specific label and not for an unspecific background signal of tannic acid.

High magnifications in TEM further depict that the intense tannic acid label is heterogeneously distributed and found between bright areas of the interstitial interface (Figure 4d). It can be further recognized that the label for tannic acid is not accidently distributed but exhibits a remarkable microarchitecture on fibrillar structures in form of fine grains (Figure 4d′,d″).

**Figure 5:**
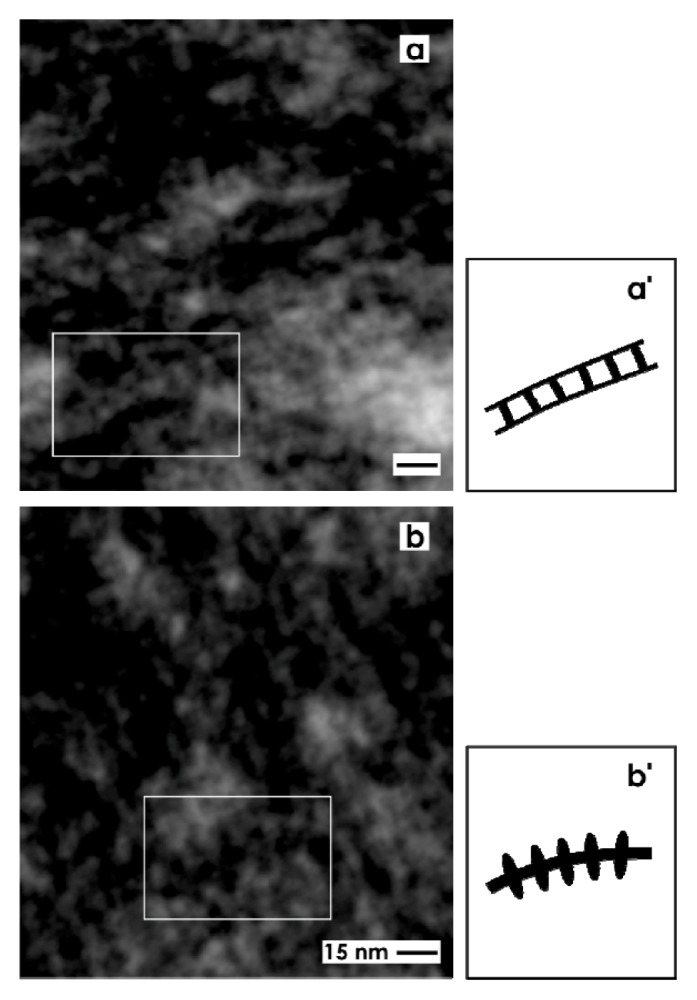
Transmission electron microscopy (TEM) of the interstitial interface within the renal stem/progenitor cell niche after fixation of specimens by GA including tannic acid. (**a**,**b**) TEM and (**a**′,**b**′) schema depict that the tannic acid label consists of (**a**) ladder- and (**b**) snow chain- like structures. (**a**) The ladder-like compounds exhibit numerous rungs with a distance of about 13 nm between each other, while the (**b**) snow chain-like structures contain vertically aligned reinforcements with a distance of about 7.5 nm between each other.

In addition, very high magnifications in TEM illuminates that the tannic acid label at the interstitial interface within the renal stem/progenior cell niche consists, as well, of ladder- (Figure 5a) as snow chain- (Figure 5b) like structures. The ladder-like compounds exhibit numerous rungs with a distance of about 13 nm between each other (Figure 5a,a′). In contrast, the snow chain-like structures contain vertically aligned reinforcements with a distance of about 7.5 nm between each other (Figure 5b,b′).

#### 2.3.5. Site-Specific Reaction of Contrasting

The label detected after fixation of specimens in GA containing cupromeronic blue (Figures 3b,4b–b″), ruthenium red (Figures 3c,4c–c″) or tannic acid (Figures 3d,4d–d″,5) is a special feature of the interstitial interface within the renal stem/progenitor cell niche as it was demonstrated earlier [7].

### 2.4. Offering a Suitable Environment for Initial Repair

Regarding therapeutical application of stem/progenitor cells for the treatment of acute and chronic renal failure it is obvious that a successful therapy depends not only on an effective surgical application, but also on a powerful seeding of stem/progenitor cells so that initial steps of regeneration can take place. As illustrated in present experiments, mesenchymal and epithelial stem/progenitor cells are exposed to a unique extracellular matrix environment. It looks inconspicuously, after fixation of specimens in GA (Figures 2,3a,4a–a″), but exhibits to a high degree specifically organized extracellular matrix as is seen after fixation in GA in combination with cupromeronic blue (Figures 3b,4b–b″), ruthenium red (Figures 3c,4c–c″) or tannic acid (Figures 3d, 4d–d″,5). These results demonstrate, on the one hand, an individual separation of both types of stem/progenitor cells within the niche. On the other hand, the present experiments clearly demonstrate that the renal stem/progenitor cell niche is not an accidental accumulation of cells. Furthermore, an impressive microarchitecture exhibits that renal stem/progenitor cells are embedded within an environment guaranteeing survival and maintenance of stemness. All of these newly detected features reflect that besides morphogenetic information, development and repair of renal parenchyma obviously need support by an individual extracellular matrix. Due to this reason, it is no wonder that simple injection of stem/progenitor cells into diseased parenchyma does not sufficiently result in expected functional regeneration [5].

## 3. Experimental Program and Materials

### 3.1. Tissue Preparation

For the present eperiments 19 one-day-old male and female New Zealand rabbits (Seidl, Oberndorf, Germany) were anesthetized with ether and killed by cervical dislocation. Both kidneys were immediately surgically removed to process them for light and electron microscopy. Paraffin embedded parenchyma of human fetal kidney (gestational age between week 16 to 18) was obtained from the stock of preparations needed for the Course of Microscopic Anatomy for medical students at the University of Regensburg.

### 3.2. Light Microscopy

Five micrometer thick sections of paraffin embedded embryonic human kidney labeled with haematoxylin eosin, or semithin sections of neonatal rabbit kidney embedded in Epon and labeled with Richardson staining, were analyzed using a Leica DM 750 microscope (Leica, Wetzlar, Germany). Images were taken with a digital camera and thereafter processed with Corel DRAW Graphic Suite X5 (Corel Corporation, Ottawa, ON, Canada).

### 3.3. Transmission Electron Microscopy

In the present investigation a protocol of fixation was applied, which was originally developed for the investigation of the adult mouse tectorial membrane matrix [17] and analysis of proteoglycans in tissue-engineered cardiovascular structures [18]. Without modifications, the mentioned techniques were applied on embryonic parenchyma of neonatal rabbit kidney to visualize specific features of the renal stem/progenitor cell niche. Following solutions were used:

1. Specimens for control: 5% GA (Serva, Heidelberg, Germany) buffered with 0.15 M sodium cacodylate, pH 7.4.
2. Series with cupromeronic blue: 5% GA buffered with 0.15 M sodium cacodylate, pH 7.4. Then specimens were incubated in 0.1% cupromeronic blue (Santa Cruz, Heidelberg, Germany) and 0.1 M magnesium chloride hexahydrate (Sigma, Taufkirchen, Germany) dissolved in sodium acetate buffer pH 5.6. Counterstaining was performed with 0.5% sodium tungstate dehydrate (Sigma).
3. Series with ruthenium red: 5% GA buffered with 0.15 M sodium cacodylate, pH 7.4 + 0.5% ruthenium red (Fluka, Taufkirchen, Germany).
4. Series with tannic acid: 5% glutaraldehyde buffered with 0.15 M sodium cacodylate, pH 7.4 + 1% tannic acid (Sigma).

The period of primary fixation was for one day at room temperature. After several washes with 0.15 M sodium cacodylate the samples were postfixed in the same buffer but additionally containing 1% osmium tetroxide (Science Services, München, Germany). Then the tissue was washed with sodium cacodylate buffer and dehydrated in graded series of ethanols.

Finally the specimens were embedded in Epon (Fluka), which was polymerized at 60 °C for 48 h. Semithin and ultrathin sections were performed with a diamond knife on an ultramicrotome EM UC6 (Leica). Sections were collected onto slot grids coated with 1.5% Pioloform (Plano, Wetzlar, Germany) and contrasted using 2% uranyl acetate and lead citrate as earlier described [19]. Analysis was performed at 80 kV using an EM 902 transmission electron microscope (Zeiss, Oberkochen, Germany).

### 3.4. Amount of Analyzed Specimens

A total of 70 exactly orientated renal stem cell niches was analyzed for the present study. Performed experiments are in accordance with the Animal Ethics Committee, University of Regensburg, Regensburg, Germany.

### 3.5. Definition of Cells within the Renal Stem/Progenitor Cell Niche

In this paper the embryonic cortex of human fetal and rabbit neonatal kidney was described. In consequence, the nomenclature of previously published papers was used [15,20,21].

## 4. Conclusions

The present data illustrate that renal stem/progenitor cells are exposed to an abundant but unique extracellular matrix. As a consequence, it remains to be investigated in the future, in how far the illustrated extracellular matrix represents an environment that can be used to influence therapeutically stem/progenitor cell development. Thus, learning from nature, considering therapeutic purposes and keeping in mind the specific microarchitecture of the renal stem/progenitor cell niche, it is concluded that stem/progenitor cells need, for better survival, seeding and functional regeneration support by a suitable extracellular matrix, when implantation into diseased renal parenchyma is intended.
